# TISs-ST: a web server to evaluate polymorphic translation initiation sites and their reflections on the secretory targets

**DOI:** 10.1186/1471-2105-8-160

**Published:** 2007-05-21

**Authors:** Renato Vicentini, Marcelo Menossi

**Affiliations:** 1 Functional Genomics Laboratory, Center for Molecular Biology and Genetic Engineering, State University of Campinas, P.O. Box 6010, 13083-875, Campinas, SP, Brazil; 2 Department of Genetics and Evolution, Institute of Biology, State University of Campinas, Campinas, SP, Brazil

## Abstract

**Background:**

The nucleotide sequence flanking the translation initiation codon (start codon context) affects the translational efficiency of eukaryotic mRNAs, and may indicate the presence of an alternative translation initiation site (TIS) to produce proteins with different properties. Multi-targeting may reflect the translational variability of these other protein forms. In this paper we present a web server that performs computations to investigate the usage of alternative translation initiation sites for the synthesis of new protein variants that might have different functions.

**Results:**

An efficient web-based tool entitled TISs-ST (Translation Initiation Sites and Secretory Targets) evaluates putative translation initiation sites and indicates the prediction of a signal peptide of the protein encoded from this site. The TISs-ST web server is freely available to both academic and commercial users and can be accessed at .

**Conclusion:**

The program can be used to evaluate alternative translation initiation site consensus with user-specified sequences, based on their composition or on many position weight matrix models. TISs-ST provides analytical and visualization tools for evaluating the periodic frequency, the consensus pattern and the total information content of a sequence data set. A search option allows for the identification of signal peptides from predicted proteins using the PrediSi software.

## Background

Translation by cytosolic ribosomes generally occurs at the first AUG in the transcript. However, in eukaryotic mRNAs, efficient recognition of an AUG codon as a translation initiation site (TIS) depends on several factors, such as the nucleotide sequence that flanks the site [1-3]. There is evidence that the context surrounding the initiation codon contributes to the control of translational initiation [4]. The sequence context of the first AUG codon, in particular that part located in the untranslated region, may modulate the efficiency with which it is recognized as a translation initiation codon [5]. If the first initiation codon lies in a suitable context, protein synthesis will be started. When the context is less than favorable, most of the protein synthesis will start at the next downstream AUG codon [6]. Moreover, other structural features of the mRNA are considered important for the efficiency of the translation initiation at a specific AUG codon, such as: the proximity of AUG to the 5' end, the secondary structure upstream and downstream from the AUG codon, the leader sequence length and the multiple upstream AUG codons [1,3,7]. Recent studies indicate that start codons of a large proportion of the human and mouse mRNAs reside in evolutionary conserved local loop structures, and some of these structures may be common in mammals and important for the efficient initiation of translation [2,8]. The frequency of the nucleotides surrounding the initiation AUG (context) has been extensively analysed in sequences available in public databases [9]. The importance of a particular position in a sequence is more clearly and consistently given by the information required to describe the pattern. The information in the sequence patterns allows one to investigate how the information is distributed across the sites and to compare one site to another [10]. Statistical analyses of the AUG initiation codon context in many organisms identified a preferential nucleotide frequency in some positions around the AUG. Recent analyses have revealed variations in the initiation context between different groups of eukaryotes. Distinct inter-taxon variations in the AUG context sequences are repeatedly observed when invertebrates, higher plants and protozoa are considered separately [11]. For instance, in vertebrates, C(A/G)CCAUGG was observed to be a consensus sequence [12]. For plant genes, a consensus context was deduced as c(A/G)(C/A)CAUGGC for monocots and A(A/C)aAUGGC for eudicots [11,13].

Upstream out-frame AUG may severely affect the translation of a gene, even if surrounded by a poor context [14], suggesting that upstream AUGs may have a role in keeping the basal translation level of a gene low [5]. Recently it was demonstrated that downstream AUG codons are utilized as alternative TISs even in mRNAs with multiple strong upstream AUGs [7]. Their occurrence must correlate with the start codon context: sub-optimal context should be accompanied by a higher frequency of downstream AUGs [15]. With this mechanism, called 'leaky scanning', multiple different proteins can be obtained from the same mRNA [5]. In this sense AUGs located downstream of the major coding sequences (CDS), may play a role in generating protein diversity [2]. The usage of a closely located downstream in-frame AUG codon as an alternative TIS can result in full and N-truncated proteins that may have the same function and be targeted at the different compartments [15-17]. Since eukaryotic mRNAs frequently contain TISs in a sub-optimal context [18], the problems of polypeptide N-end heterogeneity and finding of the genuine TIS are very topical.

*In silico *determination of the sub-cellular localization of the proteins can provide information on their function, and is dependent on the correct identification of the first AUG and their potential N-terminally polymorphic forms. This translational polymorphism may serve as an important source of diversity in both cytoplasmic and organelle proteomes [15,17].

Proteins must be localized correctly at the sub-cellular level to have normal biological functions [19]. When the final destination is the mitochondria, the chloroplast, or the secretory pathway, sorting usually relies on the presence of an N-terminal targeting sequence [20]. In the secretory pathway, proteins designated for export from the cell are labelled by an N-terminal signal sequence [21]. These signal peptides are responsible for targeting proteins to the ER for subsequent transport through the secretory pathway, and the prediction of signal peptides has become an important application of genomic and proteomic investigations. There are known cases of variation in the use of alternative signal peptides, and in the majority of cases this is due to the exclusion of the signal peptide from one or more protein products of the same gene. However in other cases, this variation involves the replacement of one signal peptide by another signal. For example, a single gene encoded 48 isoforms of protocadherin using 34 different signal peptides, each encoded by its own initial exon [22]. Alternative initial exon usage is the most common mechanism for replacing one signal peptide with another.

In this paper, we present the TISs-ST (Translation Initiation Sites and Secretory Targets) web server that investigated the usage of alternative TISs for synthesis of new protein variants possibly possessing different functions. This server deployed previously annotated complete CDSs retrieved from the NCBI UniGene database [23] and the PrediSi prediction program [21] for inspection and evaluation of alternative TISs and also target prediction of the proteins encoded by this variable site. It can be useful for finding proteins that have signal peptides in the polymorphic form, for assisting research by evaluating alternative coding potentials for eukaryotic mRNAs, and in designing synthetically created genes, especially for maximizing the translational level of an interesting protein. TISs-ST uses user-specified sequences and an optional position weight matrix (PWM) model, derived computationally from a subset of the NCBI UniGene data set, to infer the consensus around the AUG sites. This subset only includes sequences previously annotated as complete CDS. The program determines the consensus sequence and the total information content around the AUGs in five situations: (i) first transcript AUG, (ii) second in-frame downstream AUG, (iii) second out-frame downstream AUG, (iv) all other in-frame downstream AUGs, and (v) all other out-frame downstream AUGs. This information is provided with the probability of alternative TIS based on the frequency of the AUG codons. The use of these five alternatives in the analyses can provide some advantages, mainly in the prediction of TIS originating in genes with alternative splicing. All scripts and interfaces were written in Perl and R languages. This version of program is available at TISs-ST web server [24].

## Implementation

### Description of the web server

We developed a web server named TISs-ST. Basically it can be divided into two subsystems: (i) the web interface system, which is written in the Perl and HTML languages and (ii) the background process system, which is written in the Perl and R statistical languages. The web interface subsystem mainly deals with the task of receiving information from the user and checking the validity of the data submitted. The background processing subsystem computes all the analytical and prediction tasks: extracts features from the sequences, computes and displays the consensus sequences and total information content, and predicts the signal peptides of the user sequences. We used PrediSi for the prediction of signal peptides in our implementation of TISs-ST.

The web interface allows for the easy evaluation of sequences (provided in FASTA format), for the presence of putative translation initiation sites and for the prediction of signal peptides. The TISs-ST interface is designed in such a way that the user specifies all necessary parameters in the initial page, and provides many possibilities to work with the sequence files. The content of a file can be pasted in the input window, or taken from a directory on a local computer. The user has the option of setting several parameters manually.

### Input parameters

TISs-ST takes the following input data:

(i) DNA sequence data. This contains one or several sequences in FASTA format. The sequences can be in a nucleotide format, where the first ATG codon after the first 15 base pairs will be considered to be the one that encodes the initial Methionine. Since the analysis calculates the nucleotide frequencies in a context position, if the user does not choose to run the analysis with a pre-defined PWM, the amount of sequences must be bigger in order to obtain a significant result. A representative sample data is available on the website.

(ii) An optional predefined PWM model for species or species group data sets. Currently, 32 species data sets are available on the web server (Table 1) and these data can also be grouped into 20 data sets (12 phylogenetic Class, and 8 Phylum or Division).

**Table 1:** Description of the data set available in TISs-ST using a non-redundant set of genes.

**Group type I (Phylum^***a ***^or Division^***b***^)**	**Group type II (Class)**	**Species**	**Number of Sequences**
Arthropoda^*a*^	Insecta	*Anopheles gambiae*	599
		*Bombyx mori*	274
		*Drosophila melanogaster*	8821
Ascomycota^*b*^	Sordariomycetes	*Magnaporthe grisea*	152
Bryophyta^*b*^	Bryopsida	*Physcomitrella patens*	154
Chordata^*a*^	Aves	*Gallus gallus*	3135
	Actinopterygii	*Danio rerio*	7074
		*Oncorhynchus mykiss*	367
		*Oryzias latipes*	171
	Amphibia	*Xenopus laevis*	7440
		*Xenopus tropicalis*	3336
	Mammalia	*Bos taurus*	2541
		*Canis familiaris*	322
		*Homo sapiens*	12387
		*Mus musculus*	11872
		*Ovis aries*	180
		*Rattus norvegicus*	8594
		*Sus scrofa*	603
Echinodermata^*a*^	Echinoidea	*Strongylocentrotus purpuratus*	134
Magnoliophyta^*b*^	Magnoliopsida	*Arabidopsis thaliana*	14525
		*Brassica napus*	182
		*Glycine max*	373
		*Lycopersicon esculentum*	511
		*Malus × domestica*	133
		*Solanum tuberosum*	281
	Liliopsida	*Hordeum vulgare*	334
		*Oryza sativa*	1090
		*Triticum aestivum*	315
		*Zea mays*	518
Nemata^*a*^	Secernentea	*Caenorhabditis elegans*	3051
Platyhelminthes^*a*^	Trematoda	*Schistosoma japonicum*	1093
		*Schistosoma mansoni*	135

The number of sequences represents the total of unique genes. The sequences were taken from a subset of the NCBI UniGene data set [23] (retrieved August, 2005). The taxonomic classification was based on the Integrated Taxonomic Information System on-line database [26]. For the group classification, the phylum or division, and the class were used for the taxonomic level.

(iii) A filter for the AUG sites.

(iv) An option for signal peptide prediction in the protein deduced from the submitted sequence(s). This option requires the selection of the genetic code used to translate the DNA sequences.

### Output

**Figure 1:**
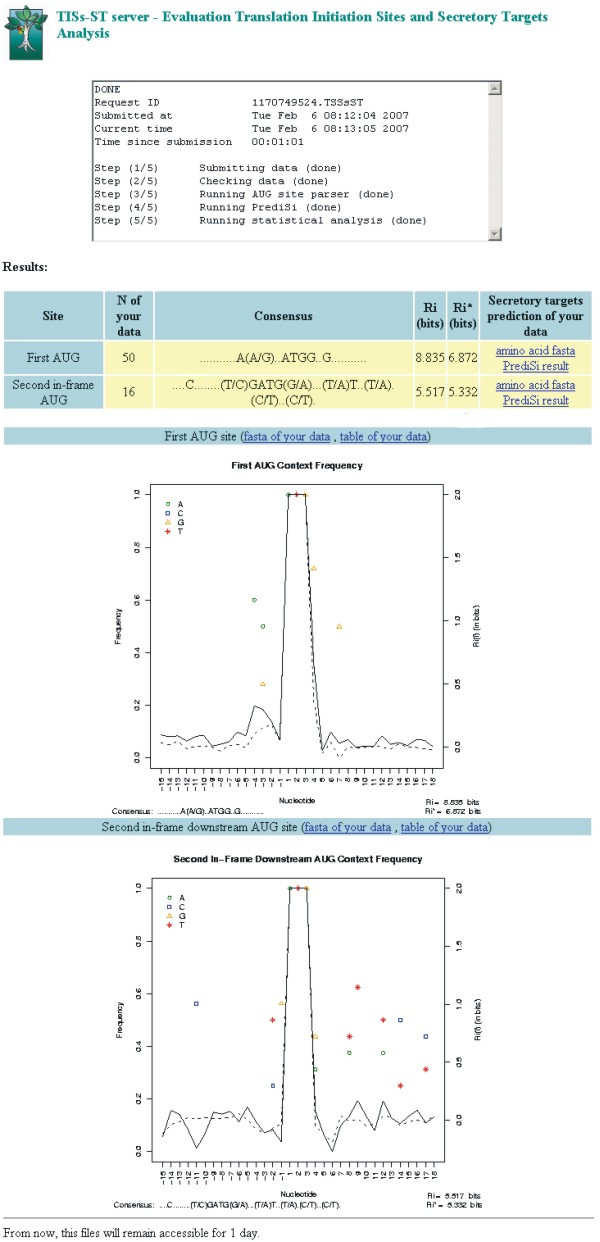
**The output interface of the TISs-ST web server**. In the TISs-ST visual output result page, every site that showed a consensus sequence had a graphic representation for the consensus and information content found. This example identified consensus for proteins encoded by first and second in-frame downstream AUG. The total information contents were 8.8 and 7.0 bits for these sites, respectively.

After submitting the data to the server, the TISs-ST program searches the consensus sequences according to the parameters selected. While the analyses are running, the web server shows a checklist of the steps finalized, from which the user can estimate the total time required for the analysis. An example of the output of the TISs-ST program is shown in Figure 1. For each site selected, the final result is a summary table with detailed information on the analysis of the data set generated. Every AUG flanking site analysed by the program is shown in a separate row of the result table, including the name of the site analysed, the number of sequences submitted, the consensus sequence found, and the total information content with or without correction for bias (measured in bits). The last column shows hyperlinks to files that include the amino acid sequences and the signal peptide prediction of the data submitted. More detailed information about a consensus sequence and the probability of localizing alternative translation initiation sites is provided by hyperlinks at the top of the graphic results.

In addition to the tabulated output, the result page shows a graphic representation of the consensus and the information content found at each site analysed. There are also hyperlinks for each sequence pattern from the user sequences, the sequence header providing information about the probability of alternative TIS based on the frequency of AUG codons, and hyperlinks to frequency tables of this pattern generated in each analysis (Figure 1).

### Graphical representation

A typical consensus graphical representation concentrates the following information into a single graph: the general consensus of the sequences; the predominance order of the nucleotides at every position (the most frequent nucleotides, in a same position, are showed before the less frequent); the relative frequencies of every nucleotide at every position; and the amount of information present at every position in the sequence [25].

TISs-ST uses two different ways to display the graphical representation of consensus sequences: (i) the information content required to describe the pattern, which is the total information content (measured in bits) at every position in a site, and (ii) a useful graphical representation for displaying the global patterns in a set of aligned sequences, to focus on the periodic frequencies of the nucleotides in the consensus pattern.

### Resources and CDS database

Non-redundant data sets of nucleotide sequences were compiled from the NCBI UniGene (retrieved August, 2005). Following the removal of sequences not annotated as 'complete CDS' (not identified previously as putatively and complete coding sequence), only sequences that had termination codon and had 15 bp before the first ATG annotated, remained in the data set. For this set, sequences were grouped according to species, based on their taxonomic classification in the Integrated Taxonomic Information System on-line database [26]. Species were also grouped according to their taxonomic level (phylum or division, and class).

### Classifying sequences into AUG site types and consensus determination

Data sets of fragments flanking the AUG were created from the -15 to +15 nucleotides of each AUG in every sequence. All fragments were grouped and the consensus sequences were determined separately for each AUG (first AUG, second in-frame downstream AUG, second out-frame downstream AUG, all other in-frame downstream AUGs, all other out-frame downstream AUGs) for each species and group of species using the 50/75 consensus rule described by Cavener [9]. The reading frame determination is based on the first AUG from the complete CDS annotated.

The consensus at a position is computed according to the following rules, with decreasing order of priority: (i) if a nucleotide at that position has a relative frequency greater than 50% and greater than twice the relative frequency of the second most frequent nucleotide, the nucleotide is given consensus status that is indicated in uppercase; (ii) if the sum of the relative frequencies of a pair of nucleotides exceeds 75%, these two nucleotides are given co-consensus status, indicated in uppercase; (iii) if there is a single most frequent nucleotide, it is given dominant status, indicated in lowercase; (iv) if two bases have the same highest frequency, they are given co-dominant status that is indicated in lowercase.

### Analysis of the information content at each position around each AUG site

The method begins by calculating a weight matrix from the frequencies of each nucleotide at each position of the aligned sequences. This matrix is then applied to the sequences to determine the sequence conservation of each individual site. Additionally we considered the nucleotide bias in genomes by using a linear noise correction [27].

A PWM model of first AUG site, called *R*_*iw *_(*b, l*), is created by using an aligned training set consisting of sequences from the nucleotides databases described before. The PWM is computed using the widely accepted information theoretical approach with some modifications [28,29]. In TISs-ST, nucleotide biases can be corrected by using the nucleotide composition observed 15 bases upstream of the start codon annotated, and the triplet noise can be corrected by using the observed frequency of each nucleotide at each position of every codon [27]. If a particular base does not appear in the data set used to create the frequency matrix, then we apply a penalty function that depends on the sample size *n *as follows: *R*_*iw *_(*b*, *l*)= 1n+2
 MathType@MTEF@5@5@+=feaafiart1ev1aaatCvAUfKttLearuWrP9MDH5MBPbIqV92AaeXatLxBI9gBaebbnrfifHhDYfgasaacH8akY=wiFfYdH8Gipec8Eeeu0xXdbba9frFj0=OqFfea0dXdd9vqai=hGuQ8kuc9pgc9s8qqaq=dirpe0xb9q8qiLsFr0=vr0=vr0dc8meaabaqaciaacaGaaeqabaqabeGadaaakeaadaWcaaqaaiabigdaXaqaaiabd6gaUjabgUcaRiabikdaYaaaaaa@30E5@[28]. Since this weight matrix is created from many sequences, it can give statistically significant evaluations of individual sites, including those used to create the matrix itself [28].

In a set of submitted sequences, we represent the *jth *sequence by a matrix *S*_*j *_(*b, l*) that contains only 0's and 1's. The individual information content of a base (*R*_*i *_(*l*)), given by some manipulation of the *R*_*i *_(*j*) [28], is the product between the base and the weight matrix:

Ri(l)=∑j=1n∑b=ATSj(b,l)Riw(b,l)n(bits per base)
 MathType@MTEF@5@5@+=feaafiart1ev1aaatCvAUfKttLearuWrP9MDH5MBPbIqV92AaeXatLxBI9gBaebbnrfifHhDYfgasaacH8akY=wiFfYdH8Gipec8Eeeu0xXdbba9frFj0=OqFfea0dXdd9vqai=hGuQ8kuc9pgc9s8qqaq=dirpe0xb9q8qiLsFr0=vr0=vr0dc8meaabaqaciaacaGaaeqabaqabeGadaaakeaafaqabeqacaaabaGaemOuai1aaSbaaSqaaiabdMgaPbqabaGccqGGOaakcqWGSbaBcqGGPaqkcqGH9aqpdaWcaaqaamaaqadabaWaaabmaeaacqWGtbWudaWgaaWcbaGaemOAaOgabeaakiabcIcaOiabdkgaIjabcYcaSiabdYgaSjabcMcaPiabdkfasnaaBaaaleaacqWGPbqAcqWG3bWDaeqaaOGaeiikaGIaemOyaiMaeiilaWIaemiBaWMaeiykaKcaleaacqWGIbGycqGH9aqpcqWGbbqqaeaacqWGubava0GaeyyeIuoaaSqaaiabdQgaQjabg2da9iabigdaXaqaaiabd6gaUbqdcqGHris5aaGcbaGaemOBa4gaaaqaaiabcIcaOiabbkgaIjabbMgaPjabbsha0jabbohaZjabbccaGiabbchaWjabbwgaLjabbkhaYjabbccaGiabbkgaIjabbggaHjabbohaZjabbwgaLjabcMcaPaaaaaa@6606@

And the total information content of the sites is the *R*_*i*_:

Ri=∑lRi(l)(bits per site)
 MathType@MTEF@5@5@+=feaafiart1ev1aaatCvAUfKttLearuWrP9MDH5MBPbIqV92AaeXatLxBI9gBaebbnrfifHhDYfgasaacH8akY=wiFfYdH8Gipec8Eeeu0xXdbba9frFj0=OqFfea0dXdd9vqai=hGuQ8kuc9pgc9s8qqaq=dirpe0xb9q8qiLsFr0=vr0=vr0dc8meaabaqaciaacaGaaeqabaqabeGadaaakeaafaqabeqacaaabaGaemOuai1aaSbaaSqaaiabdMgaPbqabaGccqGH9aqpdaaeqbqaaiabdkfasnaaBaaaleaacqWGPbqAaeqaaOGaeiikaGIaemiBaWMaeiykaKcaleaacqWGSbaBaeqaniabggHiLdaakeaacqGGOaakcqqGIbGycqqGPbqAcqqG0baDcqqGZbWCcqqGGaaicqqGWbaCcqqGLbqzcqqGYbGCcqqGGaaicqqGZbWCcqqGPbqAcqqG0baDcqqGLbqzcqGGPaqkaaaaaa@4C45@

### Prediction of secretory targets

The prediction of signal peptides was evaluated by the PrediSi prediction program [21], which allows an accurate and fast prediction of signal peptides.

### Testing the TISs-ST

#### Preparation of training and testing data

The maize data set (n = 518) and *Arabidopsis *data set (n = 14525) were used. Each of these data sets was used for training and testing the program. The total sequences were divided into two equal sets, training and testing. The training set was cross-validated by testing with the testing data set.

#### Confusion matrix method

Using the confusion matrix, various measurements of quality such as accuracy, specificity, sensitivity and the Matthews correlation coefficient (MCC) [30] were determined. In the following equation (3–6), TP refers to true positives (correctly predicted TIS), TN to true negatives (correctly predicted non-TIS), FN to false negatives (incorrectly predicted TIS) and FP to false positives (incorrectly predicted non-TIS).

*accuracy *(AC): proportion of correct predictions of the total predictions.

AC=TP+TNTP+TN+FN+FP
 MathType@MTEF@5@5@+=feaafiart1ev1aaatCvAUfKttLearuWrP9MDH5MBPbIqV92AaeXatLxBI9gBaebbnrfifHhDYfgasaacH8akY=wiFfYdH8Gipec8Eeeu0xXdbba9frFj0=OqFfea0dXdd9vqai=hGuQ8kuc9pgc9s8qqaq=dirpe0xb9q8qiLsFr0=vr0=vr0dc8meaabaqaciaacaGaaeqabaqabeGadaaakeaacqWGbbqqcqWGdbWqcqGH9aqpdaWcaaqaaiabdsfaujabdcfaqjabgUcaRiabdsfaujabd6eaobqaaiabdsfaujabdcfaqjabgUcaRiabdsfaujabd6eaojabgUcaRiabdAeagjabd6eaojabgUcaRiabdAeagjabdcfaqbaaaaa@413C@

*specificity *(SP): proportion of true negatives to the total negatives.

SP=TNTN+FP
 MathType@MTEF@5@5@+=feaafiart1ev1aaatCvAUfKttLearuWrP9MDH5MBPbIqV92AaeXatLxBI9gBaebbnrfifHhDYfgasaacH8akY=wiFfYdH8Gipec8Eeeu0xXdbba9frFj0=OqFfea0dXdd9vqai=hGuQ8kuc9pgc9s8qqaq=dirpe0xb9q8qiLsFr0=vr0=vr0dc8meaabaqaciaacaGaaeqabaqabeGadaaakeaacqWGtbWucqWGqbaucqGH9aqpdaWcaaqaaiabdsfaujabd6eaobqaaiabdsfaujabd6eaojabgUcaRiabdAeagjabdcfaqbaaaaa@37E6@

*sensitivity *(SN): proportion of true positives to the total positives.

SN=TPFN+TP
 MathType@MTEF@5@5@+=feaafiart1ev1aaatCvAUfKttLearuWrP9MDH5MBPbIqV92AaeXatLxBI9gBaebbnrfifHhDYfgasaacH8akY=wiFfYdH8Gipec8Eeeu0xXdbba9frFj0=OqFfea0dXdd9vqai=hGuQ8kuc9pgc9s8qqaq=dirpe0xb9q8qiLsFr0=vr0=vr0dc8meaabaqaciaacaGaaeqabaqabeGadaaakeaacqWGtbWucqWGobGtcqGH9aqpdaWcaaqaaiabdsfaujabdcfaqbqaaiabdAeagjabd6eaojabgUcaRiabdsfaujabdcfaqbaaaaa@37E6@

MCC: This is regarded as a more rigorous measurement to evaluate the performance of class prediction methods. MCC equals 1 for perfect predictions, whilst it is zero for completely random predictions [30].

MCC=(TP∗TN)−(FP∗FN)(TP+FP)∗(TP+FN)∗(TN+FP)∗(TN+FN)
 MathType@MTEF@5@5@+=feaafiart1ev1aaatCvAUfKttLearuWrP9MDH5MBPbIqV92AaeXatLxBI9gBaebbnrfifHhDYfgasaacH8akY=wiFfYdH8Gipec8Eeeu0xXdbba9frFj0=OqFfea0dXdd9vqai=hGuQ8kuc9pgc9s8qqaq=dirpe0xb9q8qiLsFr0=vr0=vr0dc8meaabaqaciaacaGaaeqabaqabeGadaaakeaacqWGnbqtcqWGdbWqcqWGdbWqcqGH9aqpdaWcaaqaaiabcIcaOiabdsfaujabdcfaqjabgEHiQiabdsfaujabd6eaojabcMcaPiabgkHiTiabcIcaOiabdAeagjabdcfaqjabgEHiQiabdAeagjabd6eaojabcMcaPaqaaiabcIcaOiabdsfaujabdcfaqjabgUcaRiabdAeagjabdcfaqjabcMcaPiabgEHiQiabcIcaOiabdsfaujabdcfaqjabgUcaRiabdAeagjabd6eaojabcMcaPiabgEHiQiabcIcaOiabdsfaujabd6eaojabgUcaRiabdAeagjabdcfaqjabcMcaPiabgEHiQiabcIcaOiabdsfaujabd6eaojabgUcaRiabdAeagjabd6eaojabcMcaPaaaaaa@5FC7@

#### Accuracy test

Prediction can be performed at different levels of specificity and sensitivity by defining various thresholds for the final score. At each threshold, the numbers for the TPs, TNs, FPs and FNs are calculated, and based on these values parameters such as accuracy, specificity and sensitivity are determined using equations (3–5). As seen in Table 2, the prediction accuracy ranges from 0.64 to 0.95 at the different thresholds.

**Table 2:** Confusion matrix values and dependent parameters at each threshold value.

**Score threshold**	**Positives tested (259)**		**Negatives tested (5692)**		**Accuracy**	**SN**	**SP**	**MCC**
	**TP**	**FN**	**TN**	**FP**				
≥ 70	79	180	5570	122	0.949	0.305	0.978	0.320
≥ 60	105	154	5456	236	0.934	0.405	0.958	0.319
≥ 50	131	128	5309	383	0.914	0.505	0.932	0.318
≥ 40	157	102	5159	533	0.893	0.606	0.906	0.326
≥ 30	183	76	4801	891	0.837	0.706	0.843	0.291
≥ 20	209	50	4414	1278	0.776	0.806	0.775	0.274
≥ 10	235	24	3600	2092	0.644	0.907	0.632	0.225

Values in parentheses indicate the number of proteins in each set.

#### Specificity and sensitivity test

Specificity and sensitivity are two competing quality measurements for any two-classifier method. Table 2 shows the specificity and sensitivity of the TISs-ST at the different threshold levels, as well as the MCC values. At the highest specificity (0.98), the sensitivity is at 0.30, and at the highest sensitivity level (0.91), the specificity is reasonable, at 0.63. At a mid-range sensitivity level of 0.61 (with a threshold score of ≥ 40), 61% of all the positives can be predicted with only 9% FPs, and the best correlation is obtained (MCC = 0.33).

## Results and discussion

**Figure 2:**
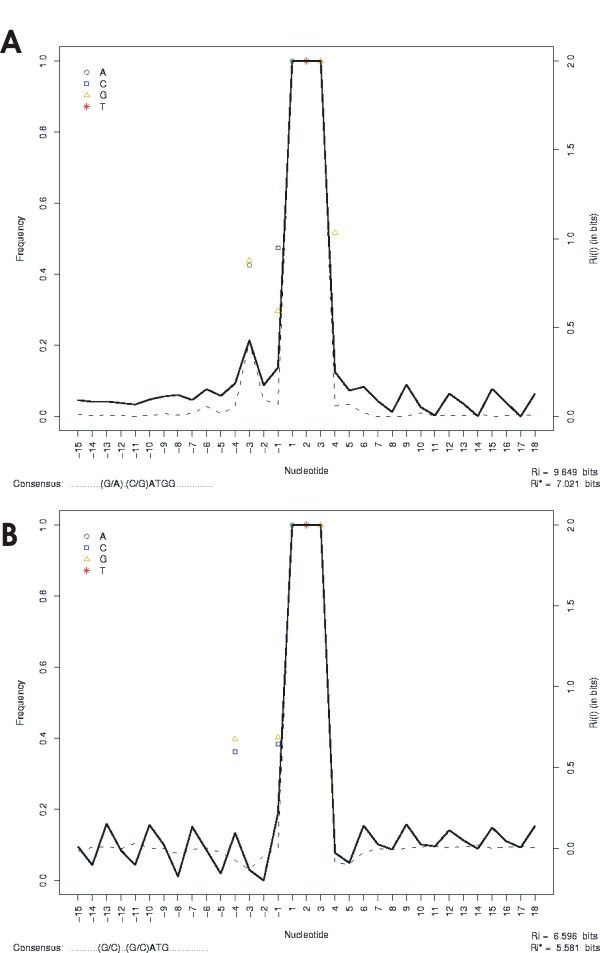
**Information content and differences in the context between two sites of *Gallus gallus***. The nucleotide sequences were selected for the determination of nucleotide frequency at positions between -15 and +15 (codon AUG corresponds to position +1 to +3). The application of two different ways of displaying the consensus sequences allows one to display the nucleotide periodic frequencies in addition to the site information content. In the analysis performed a consensus context was deduced as a"............(G/A).(C/G)ATGG.............." and "...........(G/C)..(G/C)ATG..............." for first and second in-frame downstream AUG, respectively. The total information content consisted of 9.6 and 6.6 bits for these sites. (A) First AUG site (n = 3135). (B) Second in-frame downstream AUG site (n = 1190). The frequency of each consensus base is indicated on the left Y axis, according to the 50/75 consensus rule. On the right Y axis, the lines represent the degree of site conservation measured in bits of information according to the equation given in the methods section. The continuous line is the information content without correction, and the broken line is the same information corrected for bias.

In the literature, purines are usually claimed to be important at position -3. This was the case for maize and tobacco suspension cells [11]. Although the -3 position is most conserved upstream of the start codon, experimental evidence in eudicots, showed that changes at the -2 and -1 positions affected translation efficiency at least as much as changes at the -3 position. For monocots, this effect seems to be even more pronounced because changing the C at the -1 and/or -2 position resulted in an approximately 50% reduction in translation efficiency [11]. In Figure 2, our analysis shows this topic for the chicken data set, where purines are most conserved upstream of the start codon (Figure 2A). The same did not occur for the second in-frame downstream AUG (Figure 2B).

**Figure 3:**
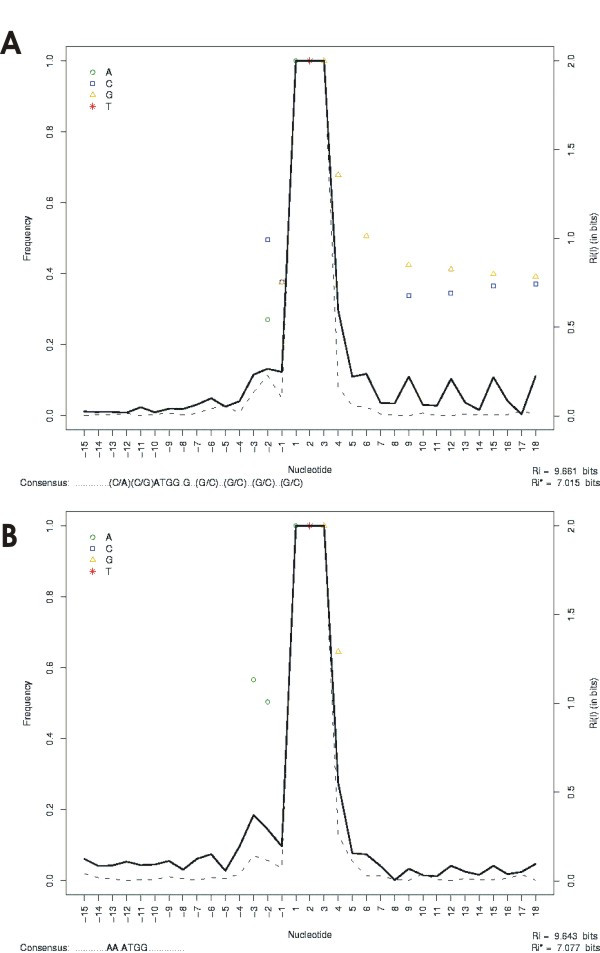
**An example of an evaluation frequency of nucleotides surrounding the initiation codon**. In the analysis performed a consensus context was deduced as a ".............(C/A)(C/G)ATGG.G..(G/C)..(G/C)..(G/C)..(G/C)" and "............AA.ATGG.............." for rice and tomato, respectively. (A) Sequence data set from *Oryza sativa *(n = 1090). (B) Sequence data set from *Lycopersicon esculentum *(n = 511).

We also performed analyses on the rice and tomato data sets, and the results of a TISs-ST search of the context surrounding the first translated AUG are shown in Figure 3. Different consensus patterns for the first AUG were found in the two data sets, and corroborate the known consensus obtained from the monocot and eudicot species [11,13].

The current method was applied to a real data set of different mRNA variants produced by differential splicing in the human phosphodiesterase 9A gene. The PDE9A gene encodes a cGMP-specific high-affinity phosphodiesterase, and the physiological implication of the isoforms produced by this gene is not yet known [31,32]. At least 21 different human PDE9A mRNA transcripts have so far been identified, which are produced as a result of alternative splicing of the 5' exons. The different PDE9A splice variants could present different translation start codons to produce the functional protein, allowing for the synthesis of a variety of polypeptides that differ in their N-terminal regions and also show differential subcellular localization [31,32].

**Figure 4:**
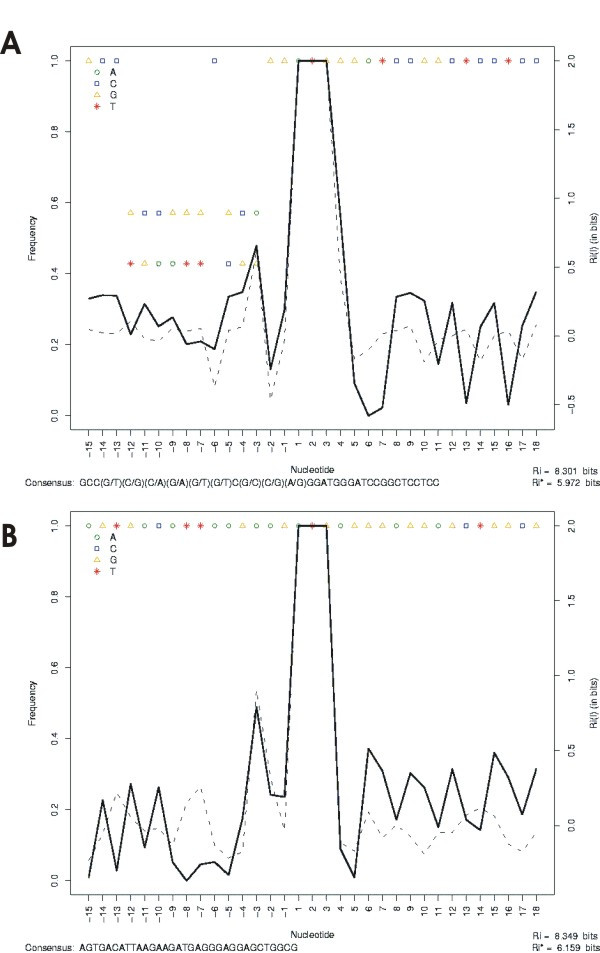
**High information content in alternative translation initiation sites of human PDE9A splice forms**. (A) The data set from the 7 isoforms that use the first start codon in exon 1. (B) The data set from the 5 isoforms that use the possible start codon present in exon 8.

We performed analyses on the data set composed of splice variants that used the first start codon in exon 1 (7 isoforms) and the possible start codon present in exon 8 (5 isoforms). The results of the analyses of these sites are shown in Figure 4. The isoforms possibly encoded by the start codon in exon 8 (Figure 4B) showed an information content (Ri = 8.3 bits) as high as that encoded by the first start codon in exon 1. This prediction corroborates the hypothesis that this AUG codon may be an alternative TIS in the splice variants of the human phosphodiesterase 9A gene [31].

The observation that sub-optimal AUG contexts are present in many genes suggests the hypothesis that this context might be involved in modulation of gene expression. This might be the case for transcripts encoding two proteins that differ at their N-terminal end [11], which would reflect in multi-targeting of the protein. Another instance where modulation of the expression by the AUG context might be important, concerns transcripts that contain a small open reading frame upstream from the main open reading frame. The main limitation of TISs-ST is that target prediction is limited to signal peptides, mainly because there are only a few free stand-alone tools available for protein sub-cellular localization prediction [33]. These free available tools do not permit one to create derivative works based on their software, or their applications are time consuming for a web access. Future work includes making the ability to predict the sub-cellular localization of proteins from the N-terminal amino acid sequence available. The analysis described above is based on the NCBI UniGene data set and the taxonomy classification of Integrated Taxonomic Information System on-line database [26]. The sequences in the NCBI UniGene data set are mostly represented by gene-specific EST clusters, and many of them are often annotated as complete CDS. This makes the determination of reading frames and the search for the 15 bp upstream of CDS, an easy step. Additional new taxonomic groups will be included in future versions. The TISs-ST local data set will be updated twice a year to incorporate future UniGene updates.

## Conclusion

Several molecular mechanisms might provide for efficient translation of the mRNAs containing upstream AUGs including leaky scanning and reinitiation or internal initiation of the translation. The contributions of these mechanisms remain uncertain but several recent studies suggest that the impact of at least some of them might be substantial [2,3,7,34-36].

Many databases in this research area are available on the web. Examples include Transterm, an information resource devoted to contexts of translation initiation and termination sites [37], and UTRdb, a curate database of 5' and 3' untranslated sequences of eukaryotic mRNAs [38]. With these databases, it is possible to obtain the structure and detect the presence of known regulatory elements in UTR sequences, and the annotation of experimentally defined functional motifs from sequence contexts of annotated translation initiation and termination codons. But currently no computational tools are available for the accurate prediction of alternative TIS, and investigations in this field could contribute to a better understanding of the complexity of mechanisms used by the cell to expand the diversity of proteins encoded by the genome. In this work we have presented a new online web server to evaluate translational variability reflected by alternative TISs. Comprehensive comparisons of contexts that surround the alternative TISs are very topical in eukaryotic mRNAs, and in addition, such translational polymorphism is a source of variability in cytoplasmic and organellar proteomes [15]. TISs-ST provides a collection of pre-analysed data sets extracted from the NCBI UniGene database, and focuses on the sequences flanking the various AUG along the complete CDSs.

## Availability and requirements

• Project name: TISs-ST

• Project home page: 

• Operating systems(s): Platform independent

• Programming language: Perl and R

• Other requirements: to build a local version of the web-service it is necessary to have a web server that allows CGI and Perl.

• License: under the GNU General Public License

## Authors' contributions

RV conceived the study, conducted the work and drafted the manuscript. MM participated in the design and coordination of the study and helped draft the manuscript. All the authors read and approved the manuscript.
